# Comparing Self-Report Pre-Exposure Prophylaxis Adherence Questions to Pharmacologic Measures of Recent and Cumulative Pre-Exposure Prophylaxis Exposure

**DOI:** 10.3389/fphar.2019.00721

**Published:** 2019-07-05

**Authors:** Jill Blumenthal, Elizabeth C. Pasipanodya, Sonia Jain, Shelly Sun, Eric Ellorin, Sheldon Morris, David J. Moore

**Affiliations:** ^1^Department of Medicine, University of California San Diego, La Jolla, CA, United States; ^2^Department of Psychiatry, University of California San Diego, La Jolla, CA, United States; ^3^Family Medicine and Public Health, University of California San Diego, La Jolla, CA, United States

**Keywords:** adherence, men who have sex with men (MSM), self-report, pre-exposure (PrEP) prophylaxis, pharmacologic measures

## Abstract

As pre-exposure prophylaxis (PrEP) effectiveness is strongly linked to adherence, we sought to determine if certain self-report measures could be used to inform objective PrEP adherence. We studied participants from the TAPIR study (a multicenter randomized study of daily text messages to support adherence to PrEP In At-Risk), a 48-week randomized controlled trial of HIV-uninfected men who have sex with men (MSM) randomized to receive text message to support adherence versus standard of care. Self-reported medication adherence was assessed using several validated measures modified for PrEP. Objective PrEP adherence was determined through dried blood spot (DBS) measurement of intracellular tenofovir diphosphate (TFV-DP) and emtricitabine triphosphate (FTC-TP). A summary of adherence was estimated using responses to the seven adherence items at weeks 12 and 48 using confirmatory factor analysis. Correlations between self-report questions and drug concentrations were estimated with Pearson’s correlations for continuous outcomes and point-biserial correlations for dichotomous outcomes. Receiver operating characteristic (ROC) analyses were conducted to assess the performance of self-report measures in predicting protective or perfect TFV-DP concentrations. Of the 369 participants who completed week 12 or 48 visits, the mean age was 35 (standard deviation 9 years), with 79% White, 12% Black, and 29% Hispanic. Correlations between self-report measures of adherence (both individual items and the adherence factor) and quantifiable FTC-TP and continuous TFV-DP concentrations showed that all self-report measures were significantly associated with these objective measures. Compared to a summary measure of self-reported adherence, the 4-week percent taken question medication recall was the only self-report item similarly or more strongly associated with recent adherence and long-term protective and perfect adherence at weeks 12 and 48. ROC analysis also showed that 4-week percent taken question had a reasonable AUC (0.798 at week 12 and 0.758 at week 48) in predicting protective TFV-DP concentrations. All single-item self-report questions assessing PrEP adherence were significantly associated with biomarker quantification, with the 4-week percent taken question performing best. Therefore, in the absence of drug concentration measurements, a 4-week self-report percent taken question may be a good single-item measure of PrEP adherence.

## Background

Over the last several years, HIV prevention has increasingly included biomedical strategies using pre-exposure prophylaxis (PrEP). Once daily tenofovir disoproxil fumarate/emtricitabine (TDF/FTC) has been shown to greatly reduce the risk of HIV infection in populations at risk for HIV acquisition (Grant et al., 2010; Baeten et al., 2012; Thigpen et al., 2012; Grant et al., 2014; Liu et al., 2016; McCormack et al., 2016). Sub-optimal adherence severely undercuts its effectiveness as an HIV prevention strategy. In men who have sex with men (MSM), four doses or more of PrEP per week has been shown to confer upwards of 99% risk reduction (Anderson et al., 2012; Desai et al., 2017). Taking less than four doses per week is considered inadequate to provide sufficient protection against HIV infection (Grant et al., 2014; Liu et al., 2016; Hojilla et al., 2018). As PrEP becomes increasingly available in clinical settings, one of the challenges for providers becomes how to accurately assess PrEP adherence outside of a research setting.

Presently, no single “gold standard” has been ubiquitously adopted to assess adherence. Research studies have employed biomarker quantification as an objective adherence measure. At present, these pharmacological assays are costly, require specialized laboratory equipment and personnel, and take time to yield results; however, there is research underway to develop point of care immunoassays in urine that could be used as clinical tools (Koenig et al., 2017;Gandhi et al., 2018). Until real-time drug concentration testing is available, biomarker quantification can be used to gauge the practicality and accuracy of alternative methods, including self-report measures. Several clinical trials have determined concentration thresholds for TFV, FTC, and their metabolites [tenofovir-diphosphate (TFV-DP) and emtricitabine-triphosphate (FTC-TP)] in various biologic mediums that correspond to recent and long-term PrEP dosing, respectively (Anderson et al., 2012; Baxi et al., 2015; Wahl et al., 2017). Unfortunately, studies analyzing the concordance between biomarker concentrations and other adherence methodologies have returned with mixed results. Device-assisted medication event monitoring system (MEMS)-caps (Musinguzi et al., 2016) is moderately correlated with drug concentrations, but they present unique technological challenges and are costly. Pill counting and medication possession ratios (i.e., pharmacy refills) have weaker concordance to electronic monitoring, as they assume all unaccounted doses were ingested and can be manipulated by patients. (Haberer et al., 2015).

Self-report adherence measures generally have the lowest concordance with drug concentrations (Amico et al., 2014; Baxi et al., 2015; Musinguzi et al., 2016; van der Straten et al., 2016). Clinical trials have often adapted self-report adherence questions from antiretroviral therapy (ART) research for use in PrEP, but the lack of a standardized method results in large variability of collecting adherence outcomes (Musinguzi et al., 2016). Moreover, subjective adherence reporting is fundamentally flawed. In particular, overestimation of adherence either as a result of social desirability or recall bias is common across medical disciplines and has been observed in many PrEP efficacy trials and demonstration projects (Amico et al., 2014; Musinguzi et al., 2016; van der Straten et al., 2016; Baker et al., 2018). Despite these limitations, self-report is a non-invasive, ecologically valid, low-burden method that has already been implemented in clinical settings. Further investigation into developing, adapting, and refining accurate self-report PrEP adherence measures is warranted (Haberer, 2016).

In this current analysis, we analyze the concordance between several self-report adherence questions with two pharmacologic drug level measures used in a PrEP demonstration project of MSM to identify the most accurate PrEP-appropriate self-report adherence measures.

## Methods

### Participants and Procedures

We employed a well-characterized high-risk cohort of MSM enrolled in the California Collaborative Treatment Group (CCTG) 595 TAPIR study (A Multicenter Randomized Study of Daily **T**ext Messages to Support **A**dherence to **P**rEP **I**n At-**R**isk for HIV Individuals; NCT01761643) (Moore et al., 2017). Participants from four Southern California medical centers [University of California, San Diego (UCSD); University of Southern California; LA Biomed at Harbor-University of California Los Angeles; and Long Beach Department of Health and Human Services] were randomly assigned to a daily text-messaging intervention (individualized Texting for Adherence Building—iTAB) versus standard of care to determine the efficacy of iTAB on PrEP adherence.

TAPIR participants were MSM, 18 years or older, and HIV-negative as confirmed by an antigen/antibody (Ag/Ab) assay or Ab assay plus HIV nucleic acid test. Additional eligibility criteria included having persistent elevated risk of HIV acquisition through condomless anal intercourse with HIV-positive men and/or partners of unknown status or having a recent sexually transmitted infection (STI) diagnosis. Over a 48-week study period, all participants received once-daily PrEP with TDF/FTC, risk reduction and adherence counseling, safety monitoring, and HIV/STI testing every 3 months. Data were collected at baseline, weeks 4, 12, 24, 36, and 48 by both confidential in-person interviews and computer assisted self-interview (CASI) instruments, which included a survey with self-report adherence measures. All CASI questions were answered and recorded by the participant. At weeks 12 and 48, biologic markers of adherence were measured, described in the next section. In the main study, iTAB participants were more likely to have tenofovir drug concentrations corresponding to near-perfect adherence (∼7 doses per week). This analysis includes the 369 TAPIR participants with completed study visits at weeks 12 or 48 after initiating PrEP.

### Measures

Pre-exposure prophylaxis as a biological marker of adherence was determined through dried blood spot (DBS) measurement of intracellular TFV-DP and FTC-TP (measuring predominately intra-erythrocytic concentrations). Both TFV-DP and FTC-TP were quantified at weeks 12 and 48 using a liquid chromatography–tandem mass spectrometry (LC-MS/MS) assay previously validated (Castillo-Mancilla et al., 2013). Protective TFV-DP drug concentrations were defined as >700 fmol/punch (∼4 doses per week) and perfect TFV-DP drug concentrations were defined as >1,250 fmol/punch (∼7 doses per week). FTC-TP concentrations were considered consistent with dosing in the last 48 h if they had quantifiable values.

Self-reported medication adherence was measured at all study visits after baseline using seven CASI questions, some of which were taken from the ACTG Adherence Instrument (Chesney et al., 2000) and other commonly used adherence questions (Berg et al., 2012; Wilson et al., 2014), modified to assess PrEP (**Table 1**). Each question was examined individually. We used CASI self-reported adherence data from weeks 12 and 48.

**Table 1:** Self-reported adherence questions and responses.

Type	Questions with Possible Responses (number)
4-week ability	Thinking about the past 4 weeks, how would you rate your ability to take all of your PrEP medications? Very poor, Poor, Fair, Good, Very Good, Excellent (6)
4-week frequency	Thinking about the past 4 weeks, how often did you take all of your PrEP medications? None, A Little, Sometimes, Most of the time, All of the time (5)
4-week percent taken	Thinking about the past 4 weeks, what percent of the time were you able to take all your PrEP medications? 0–100%
3-month recall	When was the last time you were not able to take your PrEP medication? Never, >3 months ago, 1–3 months, 2–4 weeks, 1–2 weeks, past week (6)
1-month good job	In the last 30 days, how GOOD A JOB did you do at taking your PrEP medication in the way you are supposed to? Very poor, Poor, Fair, Good, Very Good, Excellent (6)
1-month frequency	In the last 30 days, how OFTEN did you take PrEP medication in the way you are supposed to? Never, Rarely, Sometimes, Usually, Most always, Always (6)
1-month difficulty	In the last 30 days, how HARD was it for you to take your PrEP medication in the way you are supposed to? Never, Rarely, Sometimes, Usually, Most always, Always (6)

### Statistical Analysis

A summary of adherence was additionally estimated using participant responses to the seven adherence items at weeks 12 and 48 using confirmatory factor analysis (CFA). CFA is a structural equation modeling technique of examining the relationship between observed variables and their underlying latent constructs. Similar to other clustering methods, CFA summarizes data and estimates the amount of shared variance between a set of variables (Jackson et al., 2009). A one-factor model, accounting for the correlations among all seven PrEP questions and capturing a single latent construct of adherence to PrEP that could be considered a “purer” measure of adherence purged of measurement error, was estimated using robust maximum likelihood estimation (MLR) that is robust to non-normally distributed data (Muthén and Muthén, 1998–2015). Several indices of model fit were used to evaluate the fit of the one-factor model to the data; in particular, the Comparative Fit Index (CFI), the Root Mean Square Error of Approximation (RMSEA), and the Standardized Root Mean Square Residual (SRMR) were examined and guidelines for model evaluation were used to judge fit (i.e., CFI > 0.90, RMSEA < 0.06, and SRMR < 0.08 are generally regarded as indicating good-fitting models) (Hu and Bentler, 1999).

Associations between individual self-report questions assessing adherence and objective measures of adherence (FTC-TP and TFV-DP) were assessed using correlation analyses. The adherence factor and objective adherence measures were also correlated to obtain a measure of association between a summary measure of self-reported adherence and DBS measurements. Correlations between continuous variables were estimated as Pearson’s correlations while correlations between continuous self-report adherence measures and dichotomous DBS measurements were estimated as point-biserial correlations using robust maximum likelihood estimation (MLR). To account for the non-independence of observations due to participants’ repeated assessment, standard errors were adjusted using a Huber–White sandwich estimator (Huber, 1967; Muthén and Muthén, 1998–2015).

To identify the self-report item best assessing PrEP adherence, the relative strength of associations between individual self-report items and objective adherence were compared to the strength of association between the summary adherence factor and objective measures of adherence. To do so, correlation coefficients were first converted into *z*-scores using Fisher *Z* transformations and tests of the equality of correlation coefficients (i.e., comparisons associations between individual self-report items and biologically-quantified adherence versus the association between the summary adherence factor and biologically-quantified adherence) were carried out using asymptotic *z*-tests (Steiger, 1980; Lee and Preacher, 2013). Corrections for multiple comparisons were made using the Benjamini–Hochberg procedure at a false discovery rate of 0.25 (Benjamini and Hochberg, 1995).

Receiver operating characteristic (ROC) analyses were also conducted to assess the performance of these self-report measures in predicting protective or perfect TFV-DP concentrations (Fawcett, 2006). Area under the ROC curve (AUC) was calculated with a 95% confidence interval. An AUC of 0.5 indicates no discrimination and an AUC of 1.0 indicates a perfect diagnostic test. Therefore, consistent with current statistical consensus, an AUC of < 0.7 was considered poor, 0.7 to 0.8 adequate, and 0.8 to 0.9 very good. Factor analyses and correlational associations were carried out in Mplus v7.4 while ROC analyses were carried out using Rv3.5.1 (Muthén and Muthén, 1998–2015).

## Results

Of the 369 participants contributing data to these analyses, the mean age was 35 with a standard deviation of 9 years. The majority of participants were white (79%), with 12% Black and 29% Hispanic. More than half of the participants held a bachelor’s or advanced degree and nearly two-thirds had a monthly income of $2,000 or greater. Half were in the intervention arm of TAPIR (**Table 2**).

**Table 2:** Participant demographics and sample characteristics.

Characteristic	Descriptive statistic
Age, mean (SD)	35 (9)
Education, n (%)	
High school or Less	24 (7%)
Some college	137 (37%)
Bachelors	130 (35%)
Some post-graduate	18 (5%)
Advanced degree	60 (16%)
Race and ethnicity, n (%)	
White	292 (79%)
Black	44 (12%)
Hispanic	105 (289%)
Income, n (%)	
<$2,000 per month	76 (21%)
≥$2,000 per month	238 (65%)
Intervention arm, n (%)	182 (49%)

The fit of a one-factor CFA composed of all self-report adherence was adequate (CFI = .946, RMSEA = .078, and SRMR = .024), and all adherence items had large and significant factor loadings, suggesting good associations with the latent adherence construct (**Table 3**) (Hu and Bentler, 1999). Correlation analyses showed significant associations between all self-report adherence questions and pharmacologic measures. Specifically, individual self-report items and the adherence factor were correlated with quantifiable FTC-TP concentrations, continuous TFV-DP concentrations, and TFV-DP dichotomized at protective (>700 fmol/punch) and perfect (>1,250 fmol/punch) concentrations. Furthermore, the largest correlations were consistently between the various self-report measures and protective levels of TFV-DP (**Table 4**). We then compared 1) the associations between self-report items and biologically quantified adherence versus 2) the association between the summary adherence factor and biologically quantified adherence. Results of asymptotic *z*-tests suggested that 4-week percent taken was more significantly associated with recent adherence (quantifiable FTC) while 4-week ability, 4-week frequency, 3-month recall, and 1-month difficulty were associated with recent adherence to the same degree as the summary measure. The remaining self-report items had significantly weaker correlations. In terms of longer-term adherence, 4-week percent taken was associated with protective TFV-DP concentrations (>700 fmol/punch) to the same degree as the summary measure; all other measures were significantly less correlated than the summary measure. With regards to perfect TFV-DP adherence (>1,250 fmol/punch), compared with the summary measure, 4-week percent taken, 4-week ability, 4-week frequency, and 3-month recall had a similar correlations. The remaining measures were marginally or significantly less correlated with perfect TFV-DP concentrations than the summary measure (**Supplementary Table 1**). When comparing the adherence measures with continuous concentrations of TFV-DP, only 4-week percent taken and 3-month recall were similarly associated to TFV-DP concentrations as the summary adherence measure. Thus, compared with a summary measure of self-reported adherence, the 4-week percent taken medication recall was the only self-report item to consistently be similarly or more strongly associated with recent adherence and long-term protective and perfect PrEP adherence at weeks 12 and 48.

**Table 3:** Factor indicators and loadings in a one-factor model of adherence.

Indicator	Standardized loading	Standard error
4-week ability	0.908***	0.027
4-week frequency	0.869***	0.027
4-week percent taken	0.842***	0.021
3-month recall (reversed)	0.551***	0.033
1-month good job	0.910***	0.022
1-month frequency	0.893***	0.027
1-month difficulty	0.651***	0.050

**Table 4:** Correlations between pharmacological and self-report adherence measures.

Adherence measures	^a^FTC-TP	^b^TFV-DP	^a^TFV-DP ≥ 700 fm/p	^a^TFV-DP ≥ 1250 fm/p
Summary self-report (adherence factor)	0.252***	0.360***	0.465***	0.262***
4-week ability	0.239***	0.317***	0.424***	0.237***
4-week frequency	0.215***	0.309***	0.384***	0.234***
4-week percent taken	0.324***	0.372***	0.439***	0.251***
3-month recall (reversed)	0.169**	0.283***	0.296***	0.239***
1-month good job	0.177**	0.305***	0.338***	0.235***
1-month frequency	0.187**	0.299***	0.380***	0.189***
1-month difficulty	0.173**	0.209***	0.272***	0.168***

**0.05>p>0.001; ***p<0.001;

FTC-TP, emtricitabine triphosphate; TFV-DP, tenofovir diphosphate; fm/p, fmol/punch.

a Correlations using dichotomous pharmacological measures of adherence are point-biserial correlations.

b Correlations using continuous pharmacological measures of adherence are Pearson correlations.

**Figure 1:**
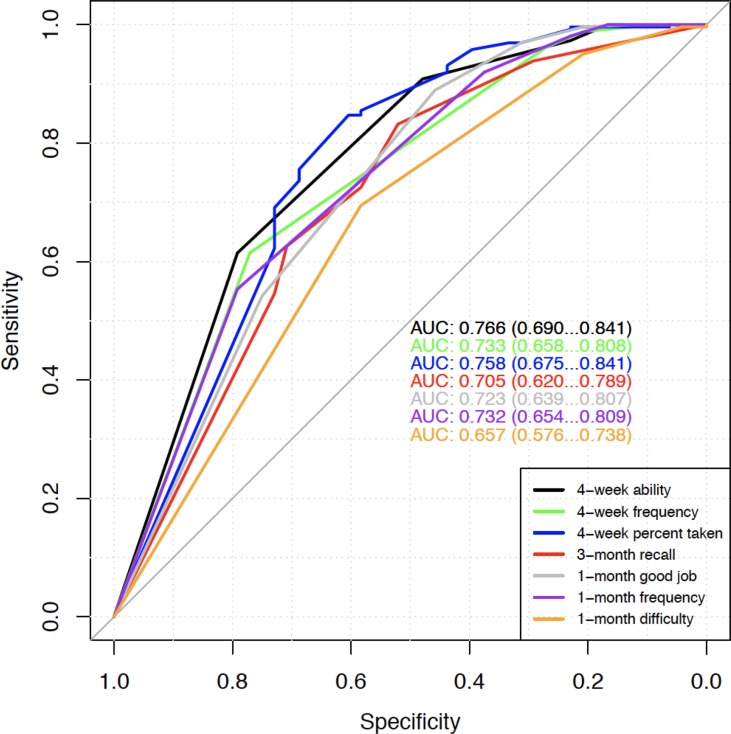
Week 48 ROC for predicting protective TFV-DP concentrations (>700 versus ≤700). AUC values with 95% confidence intervals of the seven self-report adherence questions are shown.

Finally, we evaluated the performance of the self-report questions in predicting protective or perfect TFV-DP concentrations using ROC analyses. AUC values were calculated at weeks 12 and 48. Similar to findings above, ROC analyses also showed that 4-week percent taken question had a fairly good AUC (0.798 at week 12 and 0.758 at week 48) in predicting protective TFV-DP concentrations. However, all self-reported measures were not particularly good for predicting perfect TFV-DP (AUC all below 0.7). Results from week 48 are displayed in **Figure 1**.

## Discussion

We found that all single-item self-report questions assessing PrEP adherence were significantly associated with biomarker quantification with the 4-week percent taken question performing best. In addition, the 4-week percent taken question had a reasonable AUC value in predicting protective TFV-DP concentrations at both weeks 12 and 48. Therefore, in the absence of drug level measurements, our findings suggest that a 4-week self-report percent taken question may be a good single-item measure of both recent and cumulative adherence.

Several recent studies have similarly examined different combinations of subjective and objective PrEP adherence measures and have found varying degrees of concordance among them. In the TDF2 clinical trial of men and women in Botswana, self-report adherence *via* an interview question assessing missed doses over the last 3 days was only modestly associated with quantifiable drug concentrations of tenofovir (TFV) and FTC quantified in plasma (Kebaabetswe et al., 2015). Similar results were observed in serodiscordant couples in East Africa where three types of self-reported adherence questions were not able to discriminate between steady-state daily dosing and less than steady-state daily dosing plasma TVF concentrations (Musinguzi et al., 2016). Within the preexposure prophylaxis initiative (iPrEx) trial, there were differences in consistency between self-report and PrEP drug detection by study site with good concordance in the US but large discrepancies in subjective and objective measures in non-US study sites. Self-reported recent PrEP dosing using neutral interviewing was a strong predictor of TFV quantitation in plasma in the iPrEX open-label extension (OLE) (Amico et al., 2016). Our study is an important addition to this literature because it 1) examines several self-report items assessing different aspects of adherence behavior and duration of recall, 2) employs unique statistical methods using latent constructs to develop a theoretical adherence measure without measurement errors, 3) evaluates recent and cumulative PrEP adherence using DBS quantification, and 4) includes continuous and dichotomous outcomes of objective adherence.

Because PrEP efficacy highly depends on medication adherence, it is frequently studied in HIV prevention research and evaluated in PrEP clinical care. Subjective adherence reporting is the most commonly used method to quantify adherence behavior in real time (Agot et al., 2015). As is true for self-report of medication adherence in general (Stirratt et al., 2015), there is no standard self-report adherence measure of PrEP used across research studies or clinical practice. A large number and wide variety of self-report adherence measures have already been adapted or developed to quantify PrEP adherence (Muchomba et al., 2012). As a result, it may be difficult to evaluate or compare PrEP adherence self-report in a systematic way due to lack of inconsistency in measures used. Across several large PrEP clinical trials, there was minimal overlap of self-report questions used to evaluate adherence (Amico et al., 2014; Agot et al., 2015; Kebaabetswe et al., 2015; Amico et al., 2016; Musinguzi et al., 2016). Differences in how questions are framed, what period is used, and which response options are offered may affect how adherence is reported and thus measured. Having a least one self-report measure of adherence that is widely utilized and perhaps recommended in guidelines or expert options could improve research findings and clinical outcomes through harmonization of subjective adherence assessment.

PrEP delivery in limited resource locations necessitates inexpensive and easy approaches to offer and monitor PrEP. As self-report may be the sole method to measure PrEP adherence, a question that is both sensitive and specific could offer an accurate appraisal of adherence and help direct resources to those needing additional adherence support in well-resourced but high-volume PrEP clinics. Using a single question that elicits a response best reflecting real adherence behavior is an efficient way to gauge adherence. In addition, one adherence question could be included in clinic intake forms that patients can answer more privately, potentially reducing social desirability bias (Bowling, 2005).

While our study had several strengths including a large sample size and assessments performed at multiple time points, there are some limitations. Since our study included only MSM in a resource-rich setting, our results may not be generalizable to other at-risk populations or those in resource-limited settings taking PrEP. Study timing may have significantly influenced our findings, as it was conducted soon after the FDA approval of PrEP. We had many early adopters who had high overall adherence based on DBS concentrations and were less likely to overestimate adherence. In addition, we only used single-item adherence questions to evaluate associations with PrEP drug concentrations. Some questions were designed to be asked as part of a group so alone they may have less intrinsic value. Finally, there are other commonly used self-report questions, which may be similarly or better correlated with biological measurements, not included in our study.

## Conclusions

Self-report questions to measure PrEP adherence are commonly utilized in clinical research and may be the only method deployed to assess medication adherence. It is essential to better understand which subjective measures are most accurate and how they can effectively be integrated into PrEP research and clinical care. Our findings demonstrate that a 4-week percent taken question of medication recall may best reflect true recent and longer-term adherence behavior. In the future, we will explore combinations of different self-report adherence questions that may yield even stronger associations with PrEP drug concentrations.

## Ethics Statement

This study was carried out in accordance with the recommendations of the UCSD Human Research Protections Program with written informed consent from all subjects. All subjects gave written informed consent in accordance with the Declaration of Helsinki. The protocol was approved by the UCSD Human Research Protections Program.

## Author Contributions

JB, DM, and SM contributed to the conception and design of the study; EE organized the database; SJ and SS performed the statistical analysis; JB wrote the first draft of the manuscript; EP and EE wrote sections of the manuscript. All authors contributed to manuscript revision, and read and approved the submitted version.

## Funding

The work was supported by award EI11-SD-005 from the California HIV/AIDS Research Program, award IN-US-276-D036 from Gilead and 1KL2TR0001444 to Dr. Blumenthal.

## Conflict of Interest Statement

The authors declare that the research was conducted in the absence of any commercial or financial relationships that could be construed as a potential conflict of interest.
