# Supplementary figures and images for: Comparing Self-Report Pre-Exposure Prophylaxis Adherence Questions to Pharmacologic Measures of Recent and Cumulative Pre-Exposure Prophylaxis Exposure
Source: Front Pharmacol. 2019 Jul 5;10:721. doi: 10.3389/fphar.2019.00721 (PMC6624646; doi:10.3389/fphar.2019.00721)

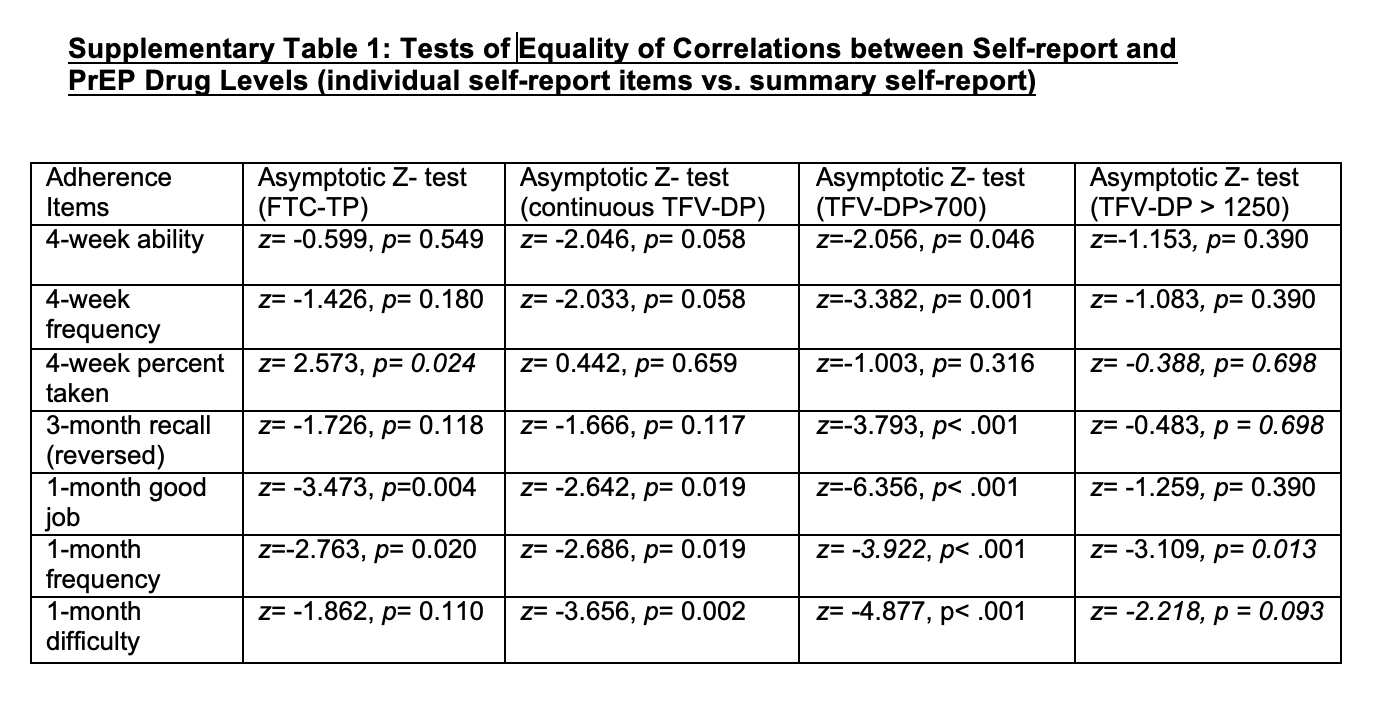

Supplement: Supplementary file 1 [file Image_1.jpeg]
